# Targeting Interleukin-17 as a Novel Treatment Option for Fibrotic Diseases

**DOI:** 10.3390/jcm13010164

**Published:** 2023-12-27

**Authors:** Margherita Sisto, Sabrina Lisi

**Affiliations:** Department of Translational Biomedicine and Neuroscience (DiBraiN), Section of Human Anatomy and Histology, University of Bari “Aldo Moro”, 70124 Bari, Italy; sabrina.lisi@uniba.it

**Keywords:** IL-17, fibrosis, autoimmune, epigenetics, biological drugs

## Abstract

Fibrosis is the end result of persistent inflammatory responses induced by a variety of stimuli, including chronic infections, autoimmune reactions, and tissue injury. Fibrotic diseases affect all vital organs and are characterized by a high rate of morbidity and mortality in the developed world. Until recently, there were no approved antifibrotic therapies. In recent years, high levels of interleukin-17 (IL-17) have been associated with chronic inflammatory diseases with fibrotic complications that culminate in organ failure. In this review, we provide an update on the role of IL-17 in fibrotic diseases, with particular attention to the most recent lines of research in the therapeutic field represented by the epigenetic mechanisms that control IL-17 levels in fibrosis. A better knowledge of the IL-17 signaling pathway implications in fibrosis could design new strategies for therapeutic benefits.

## 1. Introduction

Fibrosis is a process that develops slowly and leads to tissue degeneration, with severe consequences for organs such as the heart, lung, liver, kidney, and skin [1]. In the last few years, fibrotic disorders have significantly increased and negatively impacted public health [2]. It is estimated that in the industrialized world, 45% of all deaths can be attributed to diseases where fibrosis plays a major etiological role [2,3]. Interestingly, in pathological disorders that are based on inflammatory processes, altered repair mechanisms can lead to the formation of fibrotic tissue upon wound healing [4], which may be responsible for aberrant tissue repair [1]. During the fibrotic process, an excessive accumulation of extracellular matrix (ECM) components occurs; collagen, fibronectin, and hyaluronic acid are released and synthesized to a greater level at the site of tissue injury, leading to organ failure and death [5].

In recent years, growing evidence has highlighted that aberrant fibrosis is also a major pathological feature of many chronic autoimmune diseases, including scleroderma, rheumatoid arthritis (RA), Crohn’s disease, systemic lupus erythematosus (SLE), and Sjögren’s syndrome (SS) [6,7]. These fibrotic diseases have, in common, a persistent inflammatory stimulus based on lymphocyte-monocyte interactions that produce growth factors and fibrogenic cytokines, inducing the deposition of connective tissue components that progressively destroy the healthy tissue structure [6,7]. These data confirmed that cytokines drive the acute and chronic inflammatory responses that culminate in fibrosis activation [8]. Recently, IL-17, a pro-inflammatory cytokine, has received growing attention derived from published results collected from the study of the correlation between inflammation and autoimmune diseases [9,10,11]. Based on this evidence, herein we review recent discoveries on the role of the members of the IL-17 family in the crucial events of organ fibrosis.

## 2. The IL-17 Cytokines Family and Its Receptors

The IL-17 family is a recently identified system of secretory regulatory peptides that show homology in amino acid sequences, including an extremely preserved cysteine-knot fold structure [12,13]. IL-17A was first identified in 1993 [14] and named human cytotoxic T lymphocyte-associated antigen 8 (CTLA8). It was subsequently termed IL-17 in 1995 [15] and, more recently, IL-17A. The IL-17A gene is inserted on the 6p12 chromosome, and human IL-17A is a homodimeric protein of 35 kDa that shares a different glycosylation [15,16]. Five other members of the IL-17 family were known: IL-17B, IL-17C, IL-17D, IL-17E (also named IL-25), and IL-17F [16,17,18]. IL-17A and F are the closest members, with 50% homology, followed by IL-17B (29%), IL-17D (25%), and IL-17C (23%); IL-17E displays the lowest degree of sequence conservation (16%), implicating that IL-17E is the most dissimilar protein [19,20]. The functions of these five proteins moderately overlap with those of IL-17A, whose precise role in health and disease remains elusive. IL-17, through its binding to the IL-17 receptors (IL-17Rs), is involved in chronic and persistent inflammation, autoimmunity, and the maintenance of epithelial layer integrity [21]. These receptors share a unique protein-protein interaction domain in their cytoplasmic tail called the SEF/IL-17R (SEFIR) domain [22]. Among the IL-17R family members, IL-17RA is the best-known receptor [23,24,25]. The IL-17R is a heterodimeric complex that is formed by the IL-17RA, IL-17RB, IL-17RC, IL-17RD, and IL-17RE, with the IL-17RA subunit in association with other subunits. IL-17A and IL-17F link to a dimeric IL17RA/RC system; IL-17B and IL-17E link to a dimeric 17RA/RB system; and IL-17C links to the IL17RA/RE complex [26]. In addition, IL-17D was recently reported to bind CD93 [27]. IL-17RA and IL-17RC, acting through the binding of the SEFIR domain with the ubiquitin ligase Act1, activate the TRAF6/TAK1/NF-κB pathway and the TRAF6/TAK1/MAPK/AP1 pathway [28]. Because IL-17RB, IL-17RD, and IL-17RE also contain a SEFIR domain, a similar mechanism of activation is probable. Moreover, a conserved intracellular subdomain homologous to Toll-IL-1R (TIR) domains was revealed which could be essential for signaling downstream of the IL-1 receptor and Toll-like receptors (TLRs) [24,29]. 

## 3. Production and Functions of the IL-17 Family Members

Functionally, IL-17 cytokines, critical for normal host immune responses, are potent drivers of inflammatory responses and cancer. Both in humans and in mice, IL-17 cytokines are produced by a broad spectrum of cell types that operate on multiple cellular targets [28,30,31], triggering the secretion of pro-inflammatory cytokines, chemokines, and prostaglandins [28,32]. In this context, IL-17A has been implicated in the pathogenesis of many disorders characterized by inflammatory complications, including cardiovascular and neurological diseases [33]. IL-17A is produced by CD4^+^ and CD8^+^ T cells and γδ T cells, and it acts on endothelial cells, macrophages, fibroblasts, osteoblasts, and chondrocytes [33]. This stimulation by IL-17A enhances the production of pro-inflammatory proteins from monocytes, such as TNF-α, IL-6, IL-1β, and IL-23. IL-17A also operates on mesenchymal cells derived from synovium and skin to stimulate the production of chemokines, thus involving neutrophil (IL-8/CXCL8), lymphocyte (CCL20), and macrophage recruitment [25]. However, if dysregulated, IL-17A responses can promote the development and chronicity of inflammatory disorders in a number of autoimmune diseases [34,35]. IL-17B was demonstrated to be highly expressed during the intestinal inflammatory state and is able to induce neutrophil migration upon intraperitoneal administration, suggesting a pro-inflammatory key role [18,36,37]. Interestingly, enhanced IL-17B levels are linked with poor prognosis in patients with various types of cancer, like breast, lung, and pancreatic, reinforcing the clinical relevance of this finding [38]. IL-17B expression was also detected in synovial tissues from patients with RA, where it is mainly produced by neutrophils and chondrocytes [39]. Likewise, IL-17A and IL-17C, produced by epithelial cells and immune cells, promote anti-microbial protective activity in the skin and in the intestine [28,40,41]. In addition, IL-17C is also secreted by keratinocytes and cutaneous neurons, but in a specific condition, represented by the reactivation of the herpes simplex virus [42]. IL-17D is the least understood of the IL-17 family of proteins. It is expressed in a broad variety of healthy tissues and has been found to be expressed at high levels in immunogenic cancer cells compared to poorly immunogenic tumor cells, leading to immune rejection mediated by NK cells. Therefore, it is well documented that IL-17D provokes exacerbated viral infections [43,44]. Some interesting studies have highlighted that IL-17D stimulating the endothelial cells promotes severe pro-inflammatory cytokine activity that leads to IL-6, IL-8, and GM-CSF secretion [43,45]. IL-17E, also known as IL-25, is involved in the pathogenesis of fungal infections, allergies, and autoimmune disorders. IL-17E is diverse from other proteins of the IL-17 family; in fact, it was considered a “mucosal barrier” molecule that confers immunity against parasitic infections. Indeed, large levels of IL-17E are secreted following infection with the parasitic helminth Nippostrongylus or Aspergillus [28]. Therefore, IL-17E induces expression of IL-4, IL-5, and IL-13, all of which are associated with type 2 immunity [46], and is able to promote epithelial-cell hyperplasia by increasing mucus secretion and hyperreactivity of the airway epithelium [47].

## 4. Role of IL-17 in Fibrotic Evolution

**Figure 1:**
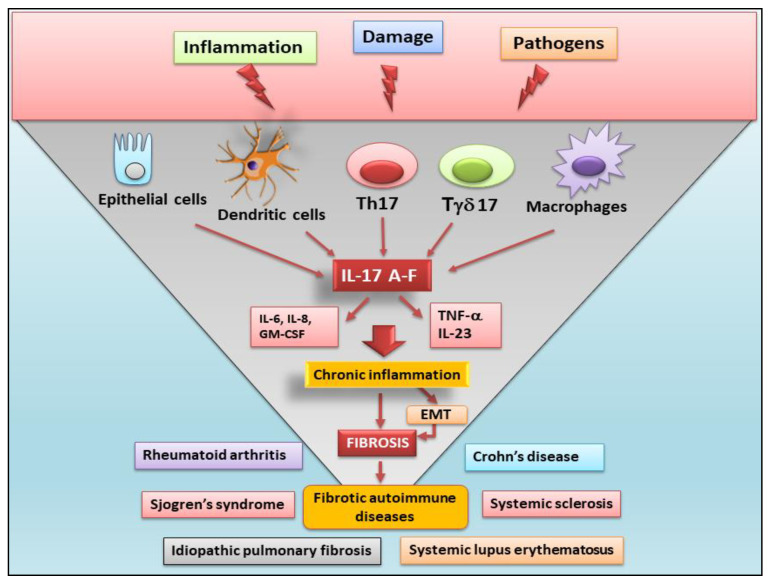
Role of IL-17 cytokines in fibrotic autoimmune diseases IL-17 is produced by epithelial cells, dendritic cells, macrophages, CD4 T helper 17 (Th17), and gamma/delta T cells (Tγδ17 cells). IL-17 signaling promotes the production of pro-inflammatory factors such as granulocyte-macrophage colony-stimulating factor (GM-CSF), tumor necrosis factor (TNFα), and the release of pro-inflammatory cytokines such as IL-6, IL-8, and IL-23. These pathogenic factors exacerbate chronic inflammation and, often through the epithelial-mesenchymal transition (EMT) process, cause the fibrotic evolution of autoimmune diseases.

All fibrotic tissues exhibit characteristics of chronic immunologically-mediated inflammatory status during the initial periods of their development. IL-17 is expressed in an altered manner in several autoinflammatory diseases. Indeed, in inflammatory chronic conditions such as liver cirrhosis, idiopathic pulmonary fibrosis, and heart failure, IL-17 contributes to the severe fibrotic process through various mechanisms, including the induction of resident stromal cells and the progression of the inflammatory status. In liver fibrosis, IL-17, operating in synergy with IL-1β, IL-6, and IL-23, continues the inflammatory process by promoting transforming growth factor-β (TGFβ) expression in hepatic stellate cells [48,49]. IL-17 stimulates the hepatocytes, involved in fibroblast activation and collagen release, to secrete periostin [50]. Accordingly, inactivation of IL-17 signaling in hepatocytes diminished the fibrotic process in the liver of murine models affected by hepatitis-induced liver injury [51]. Therefore, IL-17 is markedly expressed in the bronchial mucosa of patients affected by severe asthma and drives the epithelial-to-mesenchymal transition (EMT) process when used to induce human small airway epithelial cells in in vitro cultures [52]. Supporting the involvement of IL-17 in EMT-dependent fibrosis, Sisto et al. recently demonstrated that IL-17, through the support of IL-22, contributes to triggering the EMT-dependent fibrotic process in healthy human salivary gland epithelial cells, clarifying the role of IL-17 in the fibrotic evolution observed in SS [53]. It is interesting to note that, in patients with idiopathic pulmonary fibrosis (IPF), Th17 cells secrete TGFβ and IL-17A at high levels; in addition, when lung fibrosis was induced in vitro through bleomycin (BLM) treatment of a murine model or when Th17 cells were cultured simultaneously in the presence of human lung fibroblasts, an increase in collagen deposition and other ECM factor production was revealed [54]. Indeed, blocking IL-17 improves the fibrotic condition in the lung of murine models affected by pulmonary disease following a post-bone marrow transplant [54]. Finally, IL-17 secreted by γδ T cells and Th17 cells plays a key role in several conditions of heart fibrosis, probably involving various inflammatory events in these organs. In fact, IL-17 induces cardiac myofibroblast transformation in murine models, in which ischemia causes heart injury, as well as in experimental models of hypertension [55]. Blockade of the IL-17 signaling pathway reduces cardiac fibrosis and improves myocardial contractile function [56]. A schematic representation of the role of IL-17 in autoimmune-related fibrosis is reported in Figure 1.

## 5. Fibrosis Mediated by Several IL-17 Family Members 

Dysregulated expression of IL-17 cytokines contributes to the triggering and exacerbation of fibrosis in a variable way, depending on the specific IL-17 family member. In this paragraph, we report the most recent knowledge regarding the role of IL-17 family members in the fibrotic evolution of inflammatory diseases.

### 5.1. IL-17A

Studies conducted using experimental animal models have demonstrated the role of IL-17A in regulating the complex interplay between lung inflammation and fibrosis. After BLM treatment to induce injury, IL-17A expression is upregulated, determining the release of pro-inflammatory cytokines and chemokines by endothelial cells and epithelial cells [54,57]. These factors recruit several types of inflammatory cells to the alveolar surface, and the resulting inflammation activates pulmonary fibrosis [58]. In addition, the same situations of inflammation, neutrophilia, and pulmonary fibrosis develop after IL-17A production following IL-1β treatment [54]. Confirming the pro-fibrotic role of IL-17A, blocking IL-17A through the intraperitoneal administration of an antibody against IL-17A reduces the acute inflammatory and fibrotic features in an experimental animal model [58]. Consequently, it is not surprising that the depletion of alveolar macrophages decreased the effects of IL-17 on the activation of lung fibrosis, supporting the hypothesis that these cells are involved first in the activation, producing pro-fibrotic factors such as IL-1β and IL-23 [59]. The IL-17A receptor is also ubiquitously expressed on the membrane surface of epithelial cells and fibroblasts; these cells are involved in the EMT-mediated pulmonary process correlated with pulmonary fibrosis; furthermore, these cells regulate fibroblast transformation into myofibroblasts, increasing extracellular matrix deposition [60]. Recently, it has been proposed that the IL-17A-mediated signaling and EMT of intrahepatic biliary epithelial cells are involved in the pathogenesis of primary biliary cirrhosis (PBC). This study demonstrated increased protein levels of the IL-17A receptor in intrahepatic biliary epithelial cells, and the IL-17A resulted in accumulation around those cells in the patients affected by PBC [48].

Additionally, IL-17A can act as a pro-fibrotic interleukin by suppressing autophagy in epithelial cells [59], although whether autophagy has a protective effect or not is yet to be determined. In addition, in some studies, after BLM treatment, an overexpression of IL-17R in fibroblasts was detected; furthermore, the addition of exogenous IL-17 can accelerate fibroblast proliferation, accompanied by an increased synthesis of specific proteins such as α-smooth muscle actin (α-SMA) and collagen [60]. The IL-17 stimulation of fibroblasts occurs, probably, via activation of NF-κB through the NF-κB activator 1 protein (Act1) [61], a critical mediator of IL-17 receptor family signaling, especially in autoimmune conditions [62].

### 5.2. IL-17B 

Research on IL-17Bs role in fibrotic evolution has been limited, and in general, the function of IL-17B has not been thoroughly clarified. However, several studies support the possibility that the effects of IL-17A and IL-17B are very similar in the regulation of inflammation and fibrosis [63]. For example, IL-17B up-regulates the production of IL-6, IL-23, and IL-1α in the peritoneal neutrophils, macrophages, and lymphocytes [61]; in addition, it determines TNF-α and IL-1β release by the human monocyte/macrophage cell line [64]. IL-17B promotes the recruitment of cells that express the chemokine receptors CXCR4 or CXCR5, and the experimental intraperitoneal administration of recombinant human IL-17B determines the chemoattraction of neutrophils, which release chemoattractants for other cells [65]. Additionally, IL-17B can synergize with IL-33 to regulate T-helper (Th)-mediated immune responses [66]. These pro-inflammatory functions suggest that IL-17B may influence the progression of fibrosis, which follows the early stages of inflammation. This hypothesis was confirmed by the research group of Yang, who recently reported that the expression of IL-17B was affected by dysbiosis; this situation induces lung fibrosis by interacting with TNF-α to stimulate the secretion of Th17-cell-promoting genes and neutrophil-recruiting genes [67].

### 5.3. IL-17C

IL-17C has been shown to be expressed in CD4^+^ T cells, dendritic cells (DCs), macrophages, and epithelial cells, which produce this interleukin during antimicrobial activity [68,69], determining an enhanced inflammatory response [68,69]. The activity of IL-17C occurs through binding to the IL-17 receptor complex, consisting of IL-17RA and IL-17RE subunits [70]. IL-17RE, in particular, was expressed mainly on epithelial cell surfaces and Th17 cells. Th17 cells react to stimulation with IL-17C, producing IL-17A and IL-17F, confirming that IL-17C might regulate the initial phase of the development of inflammation [68,69]. Once again, IL-17C production is dependent on NF-κB/Act1 activation, determined by IL-17C binding to the receptor complex IL-17RA/IL-17RE and, in turn, MAPK signaling molecules expression [70,71]. The function of the IL-17C isoform in the progression of fibrosis was mainly explored in IPF [72], using a lipopolysaccharide-induced lung injury as a model of IPF. Data collected on epithelial cell damage, the release of pro-inflammatory factors, and neutrophil recruitment mediated by the release of IL-17C confirmed the key role of IL-17C in lung inflammation. IL-17Cs role in Haemophilus influenzae and cigarette smoke-induced lung inflammation has been recently reported [73], mediating the expression of neutrophilic cytokines, the recruitment of neutrophils, and lung fibrotic damage [73]. 

### 5.4. IL-17D

IL-17D has a more limited expression and is detected in B lymphocytes and resting CD4^+^ T cells; IL-17D acts, in particular, on endothelial cells, regulating their secretion of pro-inflammatory factors [74]. However, knowledge of the role of IL-17Ds in the exacerbation of pulmonary fibrosis remains poorly investigated and needs clarifying studies.

### 5.5. IL-17E 

**Figure 2:**
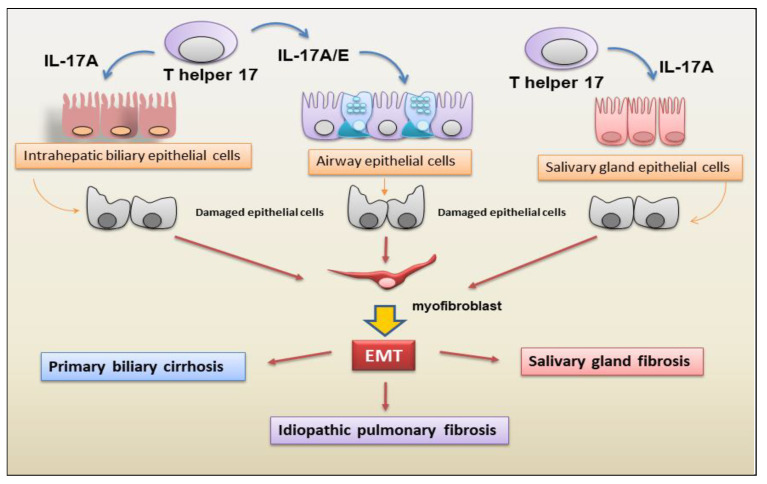
The main cellular sources and targets of IL-17A and IL-17E in the EMT-mediated fibrotic evolution of autoimmune diseases. IL-17A/E contributes to idiopathic pulmonary fibrosis, primary biliary cirrhosis, and salivary gland fibrosis through the activation of EMT, which leads to fibroblast proliferation and differentiation into myofibroblasts. EMT (epithelial-mesenchymal transition); T h17 cells (T helper 17 cells).

IL-17E, recently correlated with pulmonary fibrosis, was secreted by Th2 cells, epithelial cells, endothelial cells, T cells, alveolar macrophages, DCs, eosinophils, and basophils [75]. Xu and collaborators [76] not only demonstrated an increase in IL-17E secretion but also the activation of an EMT program in alveolar epithelial cells, determining EMT-dependent fibrosis activation in patients with IPF. These observations were confirmed by Hams [77] through the demonstration of increased levels of IL-17E in the lungs of IPF patients, which is correlated, interestingly, with IL-13 release that exacerbates collagen deposition during the IPF process. A schematic representation of the correlation between IL-17 subtypes A and E and EMT-dependent fibrosis is shown in Figure 2.

### 5.6. IL-17F

IL-17F shows a high homology of sequences and activities that mostly overlap with those of IL-17A [78]. The cells that produce IL-17F are the same as those releasing IL-17A. IL-17F is able to trigger the release of IL-6 and CXC chemokines from tracheal epithelial cells, inflammatory cells, endothelial cells, and fibroblasts, supporting the hypothesis that IL-17F could modulate autoimmune and inflammatory diseases [79,80]. However, the field of investigation into the pro-fibrotic activity of IL-17F is still pioneering.

The data related to IL-17 subtypes are reported in Table 1.

## 6. Epigenetic Regulation of IL-17 in Fibrotic Diseases

Dysregulated Th17 cell responses contribute to the immunopathogenesis of multiple inflammatory and autoimmune diseases [81]. Following aberrant activation stimuli, T helpers appear to be involved in triggering autoimmune responses against many organs, such as joints, brain, skin, gut, pancreas, salivary and lachrymal glands, and the eye. This abnormal and persistent activation leads to the onset of multiple autoimmune diseases, including multiple sclerosis (MS), psoriasis, Cröhn’s disease, type I diabetes, SS, uveitis, systemic sclerosis (SSc), and SLE [82]. It is now accepted that the fibrotic evolution of autoimmune diseases presents an excessive release of pro-fibrotic factors as a result of the activation of molecular cascades depending on chronic inflammation, and the recent challenge consists of identifying anti-fibrotic therapies that can also have value in autoimmune diseases. Previous research has significantly increased our understanding of genetic susceptibility to fibrotic diseases based on the identification of sequence variants, polymorphisms, and mutations in several genes [83,84]. However, the concordance rate for some fibrotic diseases in monozygotic twins is low, indicating that genetic predisposition is insufficient to explain disease development and suggesting a potential role of epigenetics as the missing link that connects environmental exposure to disease development [85]. The following paragraphs explore findings related to the epigenetic regulation of IL-17 in fibrosis.

### 6.1. Histone Modification and IL-17 Production

**Figure 3:**
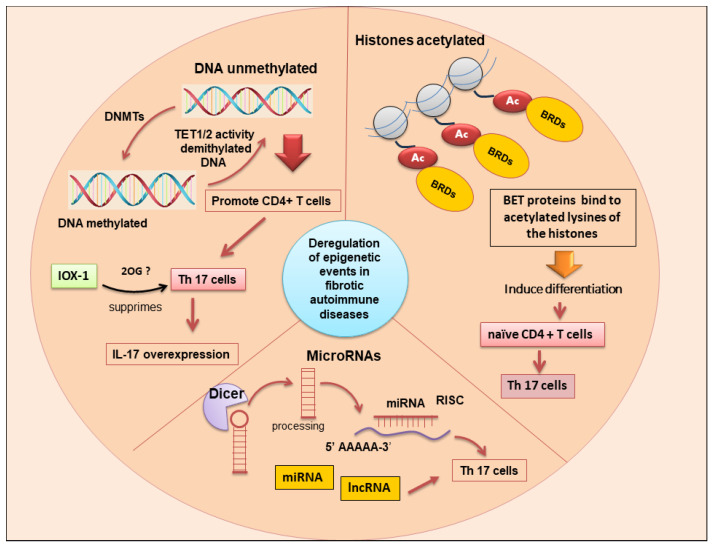
The main epigenetic changes associated with differentiation of T h17 cells and upregulation of IL-17 are DNA methylation, histone post-translational modifications, and ncRNA (lncRNA and miRNAs). Bet proteins (bromodomain and extra-terminal domain); BRDs (bromodomains); DNMTs (DNA methyltransferases); ncRNA (non-coding RNA); lncRNA (long non-coding RNA); miRNA (microRNA); Dicer (Endoribonuclease Dicer C-terminal complex); TET (ten-eleven translocation); 2OG (2Oxoglutarate Oxygenase); IOX-1 (Histone demethylase inhibitor); Risc (RNA-induced silencing complex); T h17 cells (T helper 17 cells); the symbol “?” indicates that the mechanism has not yet been clarified.

As an epigenetically modulated mechanism, differentiation of T helper cells was acknowledged to imply total changes in histone modifications such as H3K4me3 and H3K27me3 [86]. Recently, it was reported that the histone deacetylase (HDAC) inhibitor, which has potential effects on epigenetic alterations, had been shown to mitigate renal fibrotic conditions. Findings have observed the conversion of CD4^+^ forkhead box P3 (FOXP3)+ T regulatory (Treg) cells into T helper 17 cells (Th17), contributing to the progression of renal fibrosis. Worsening renal fibrosis was linked with the loss of CD4^+^FOXP3+IL-17+ T cells in splenic single-cell suspensions. FOXP3+IL-17+ T cells expressed TGF-β1 both in vitro and in vivo, and, indeed, the loss of TGF-β1 expression was confirmed using IL-17 siRNA. It is now well established that these cells play a critical role in converting Tregs into IL-17- and TGF-β1-secreting cells [87]. Therefore, targeting the epigenetic process that induces the pathogenic activation of CD4^+^ T helper cells may provide new therapeutic approaches to revolutionize the treatment of autoimmune conditions. Interestingly, removing acetyl groups by histone could occur through a series of HDACs that induce compact nucleosome structure and prevent active transcription; however, in some events, HDACs can directly activate transcription, although the exact processes by which they modulate transcription are currently poorly known [88]. In addition, HDACs seem to modulate the fibrotic process through fibroblast proliferation, senescence, and ECM production [89,90]. Based on this evidence, recent studies report that histone HDAC inhibitors can decrease the inflammatory status mediated by CD4^+^ T cells and subsequent fibrotic evolution [91,92] (Figure 3).

### 6.2. DNA Demethylation in the Control of IL-17 Pro-Fibrotic Activity

DNA demethylation, generally linked with gene silencing, has recently been identified as a strategy in the regulation of IL-17-dependent fibrosis. Ichiyama et al. demonstrated DNA methylation at T helper-specific IFNγ, IL17A, and Foxp3 [93]. Therefore, it was demonstrated that the H3K27me3 demethylase JMJD3 [94] and DNA demethylases Ten-Eleven-Translocation (TET)2/TET35 are key activators of IL-17 expression, and recent data have evidenced that H3K9me3 may also modulate the expression of IL-17 [95]. It is now accepted that bromodomain antagonists [96] and histone H3K27me3 demethylase inhibitors [97] are able to decrease the inflammatory status linked to CD4^+^ T cell activation. Emerging evidence underscores the importance of bromodomain antagonists that interfere with epigenetic events on histones related to transcriptional processes. The bromodomain and extraterminal domain (BET) family proteins, consisting of BRD2, BRD3, BRD4, and BRDT, are characterized by two bromodomains that recognize and bind to lysine-acetylated histones and other acetylated proteins with different degrees of affinity. Small-molecule BET inhibitors interact on the acetyl moiety inserted into the bromodomain acetyl-lysine-binding pocket, which is specific to the BET family proteins, and this allows BET inhibitors to be perfect candidates for blocking the constitutively active regions that have active histone marks. Indeed, BET inhibition has been shown to reduce the differentiation of naive T cells into Th17 cells [98]. Interestingly, studies in vitro have demonstrated that BET inhibition potently suppressed Th17 cell responses in explanted lung tissue from cystic fibrosis’ patients with a history of chronic lung inflammation. Thus, these BET inhibitors are able to modulate T cell responses, specifically Th17-mediated inflammation, to inhibit IL-17-driven chemokine production in human bronchial epithelial cells through processes that include bromodomain-dependent inhibition of acetylated histones at the IL-17 gene locus [99]. In addition, in a murine model of acute pseudomonas aeruginosa lung infection, BET inhibition diminished inflammatory conditions without exacerbating infection, suggesting that BET inhibitors may be a potential therapeutic candidate in patients with cystic fibrosis [100]. 

Based on this evidence, targeting Th17 cells as well as those molecules mediating the differentiation and inflammatory functions of these cells can become a plausible therapeutic approach for many autoimmune diseases. While classical biologic agents (monoclonal antibodies or recombinant proteins) targeting IL-17 and IL-23, as well as inhibitors of RORγt, have shown potent efficacy only in some fibrotic diseases such as psoriasis and RA, unfortunately, this potential has not been observed in other diseases, such as uveitis and Cröhn’s disease [101]. This has led to the identification of new potential therapeutic targets, and recently, IOX1 (a histone demethylase inhibitor) was identified as the potential candidate that suppresses Th17 function, targeting TET2 activity on the IL-17a promoter. The TET proteins TET1, TET2, and TET3 catalyze 5-methylcytosine (5 mC) conversion to 5-hydroxymethylcytosine (5 hmC) to regulate the DNA demethylation mechanism [102]. However, the potential therapeutic effect of the inhibitors of TET proteins has, until now, not been fully appreciated or developed. IOX1 is a general 2-Oxoglutarate Oxygenase (2OG) inhibitor that can also target other histone and DNA demethylases. Interestingly, IOX1 does not seem to have any direct interaction with demethylases, which could potentially activate IL-17A expression but, probably, suppress Th17 cells through targeting other 2OG enzymes [103]. From early studies conducted in the laboratory, IOX1 appears to have the advantage of similar efficacy with less cellular toxicity when compared to previously known inhibitors of IL-17-mediated inflammation, such as Tofacitinib [103] (Figure 3).

### 6.3. Correlations of Non-Coding RNA Expression and IL-17 Levels in Fibrosis

Different cascades and signaling pathways regulate fibrosis [6,104]. Recently, in addition to the large number of factors involved in fibrotic evolution, a number of non-coding RNAs (ncRNAs) have been found to affect fibrotic processes. The ncRNAs include a vast number of transcripts, within which long ncRNAs (lncRNAs) and microRNAs (miRNAs) have been extensively investigated in recent years, as they have demonstrated extensive regulatory activity on mRNA-coding genes. miRNAs are single-stranded transcripts with sizes of about 22 nucleotides, produced from precursors of 60–100 nucleotides by modifications conducted by an RNase III endonuclease, namely Dicer [105]. These small transcripts suppress the synthesis of proteins through base pairing to the 3′ untranslated region (3′UTR) of mRNA or, rarely, to the 5′UTR and coding regions [106]. When created, one or both strands of the miRNA duplex can be assimilated into the RNA-induced silencing complex regulating gene transcription [107]. On the other hand, lncRNAs have sizes greater than 200 nucleotides and represent a quantity higher than protein-coding genes [108]. With a total amount higher than that of protein-coding genes, their variability is correlated with the complexity of the organism and with the type of molecular pathway that they regulate. In fact, lncRNAs influence fundamental biological processes such as imprinting, chromosomal configuration, and enzymatic activation [109]. Many studies have revealed that miRNAs and lncRNAs are key regulators of the development of fibrotic processes, often correlated with autoimmune conditions. Probably they act on the most common fibrotic pathway, mediated by TGF-β, phosphatidylinositol 3-kinase/protein kinase B (PI3K/AKT), and Wnt/β-catenin. An example is represented by liver fibrosis, in which the activation and proliferation of hepatic stellate cells were regulated by miRNA or lncRNA that exerted their pro-fibrotic activity precisely by acting on the pathways just reported [110,111]. There is recent evidence that this pro- or anti-fibrotic activity may be mediated by a dysregulation of T helper cells with consequent variability in the release of IL-17. Positive correlations in miRNA expression and IL-17 levels have been observed in different studies related to fibrotic diseases or autoimmune diseases with a fibrotic evolution of the tissue or organs involved (Table 2). In experimental autoimmune uveoretinitis (EAU), an overexpression of miR-142-5p and miR-21 was detected to be correlated with increased IL-17 levels, but miR-182 was decreased [112]. In psoriasis, characterized by the fibrotic evolution of skin lesions, miR-1266 and miR-146, which are known to regulate IL-17A synthesis, were increased in the sera of these patients [113,114], also in association with RA [115,116]. A positive correlation was also observed in cardiac interstitial fibrosis, characterized by myocardial fibrosis, between IL-17 and lncRNA-AK081284 [117]. On the contrary, complicating the scenario, overexpression of ncRNA is accompanied by a reduction in IL-17 release in autoimmune conditions and other fibrotic diseases (Table 2), as observed, for example, in RA [116,118]. In an experimental model of autoimmune myasthenia gravis (EAMG), the administration of lentiviral miR-145 decreased EAMG disease, determining a concomitant decreased secretion of IL-17 [106]. In the prototypic fibrotic disease SSc, there is an increase in leucocytes in the skin, including primarily T cells. These T cells that are residing in the skin are in close proximity to the myofibroblasts, suggesting that they are governing their transdifferentiation [119] and may activate other immune cells in the inflammatory foci. It has been described in SSc fibroblasts that miRNA-129-5p is repressed compared with healthy control fibroblasts [120]. The authors also show that the T-cell cytokine IL-17 can increase miRNA-129-5p levels, and using siRNA to knock down IL-17 receptors in dermal fibroblasts reduced miRNA-129-5p levels. The actual target mRNA of miRNA-129-5p appears to be collagen alpha-1 [120]. This all suggests that the Th17 cells reduce collagen expression via the upregulation of the negative regulator miRNA-129-5p. Recently, Zhang and colleagues demonstrated that miR-125a-3p decreases levels of interlukin-17 and suppresses renal fibrosis via down-regulating TGF-β1 in Lupus nephritis (LN), an autoimmune disorder mediated by SLE. The condition of LN is accompanied by inflammation via a progressive suppression of kidney function, mediated by developing fibrosis [121]. In MS patients, the downregulation of miR-20b was revealed. In experimental autoimmune encephalomyelitis (EAE), primarily used as an animal model of human autoimmune inflammatory MS, miR-20b overexpression decreased disease severity by decreasing Th17 differentiation by targeting RORγt and STAT3 [122]. In the EAE model, miR-873 induced by IL-17 stimulation aggravated disease severity and increased inflammation by targeting Tumor Necrosis Factor Alpha-Induced Protein 3, or TNFAIP3 (A20)/NF-κ [123]. The same effect was obtained by the overexpression of miR-132 in the EAE [124]. Importantly, Du et al. reported that miR-326 expression correlated with MS disease severity in human patients, and in EAE mice, miR-326 regulates Th-17 cell differentiation through translational inhibition of Ets-1, a negative regulator of Th17 differentiation [125]. All these findings suggest that miRNA or lncRNA regulation and correlation with IL-17 are dependent on the fibrotic disease model (Table 2 and Figure 3).

## 7. IL-17 Inhibitors as a New Therapeutic Strategy

Given the strong pro-inflammatory role of IL-17, drugs that target IL-17 or the IL-17R are potential therapeutic candidates for inflammatory autoimmune diseases [126,127]. In 2016, anti-IL-17A monoclonal antibodies (mAbs), such as the IL-17 inhibitor secukinumab and the IL-17R inhibitor brodalumab, were both approved for the treatment of psoriasis [128]. More recently, the Food and Drug Administration has approved a novel drug, Ixekizumab, for the treatment of moderate-to-severe plaque psoriasis as well as active psoriatic arthritis. Ixekizumab is a humanized IgG4 mAb that selectively binds IL-17A and prevents interactions with IL-17R [128]. By targeting cells, it hampers the release of pro-inflammatory proteins, subsequently involving cellular components [127]. However, these drugs have unexpectedly demonstrated low efficacy in the diseases linked to IL-17, such as RA and MS [128]. Investigating in this context, Luo et al. discovered that a molecular complex containing the adaptor molecule Act1 and the tyrosine phosphatase SHP2 mediates autonomous IL-17R signaling, sustaining an intense inflammatory state. The resulting Act1–SHP2 complex is aberrantly increased in various autoimmune diseases, facilitating resistance to IL-17-directed therapy. The authors discovered that SHP2 inhibitors, as well as iguratimod, a small molecule that disrupts the Act1–SHP2 interaction, show promise in mouse models of MS and RA [129]. However, all monoclonal antibodies targeting the IL-17-IL-17R pathway and approved as treatments have several disadvantages, such as non-oral administration, poor tissue penetration, and various adverse effects as an escalation of the immune system’s inflammatory response. Indeed, intensive research is being performed to discover potent small molecules targeting the IL-17A/IL-17 RA protein-protein interaction to modulate immune responses as an attractive approach for immunotherapy [130]. These new small-molecule drugs (SMDs), which are orally bioavailable, are beneficial in terms of production cost, convenience of delivery, and potentially higher efficacy. Actually, numerous clinical trials of anti-IL-17A and IL-17RA antibodies are currently in progress [130,131].

## 8. Conclusions

IL-17 is critical for host defense, but its role in the regulation of many chronic inflammatory, fibrotic, and/or autoimmune diseases becomes more and more evident. Although the pivotal roles of IL-17 in chronic inflammatory conditions are increasingly enumerated, these new concepts are not enough to clarify the function of IL-17 in fibrosis, which often represents the evolution of autoimmune diseases. Overall, the thin differences in IL-17 cytokines and their receptors seem to influence their role in fibrotic evolution. A thriving field of research concerns the inflammatory mechanisms mediated by the activation of EMT, which may have links to fibrotic evolution. In addition, in recent years, various experimental data have demonstrated the key role of epigenetics in the genetic regulation of fibrosis [107]. Several epigenetic modifications are involved in this process, such as histone modifications, DNA demethylation, or miRNAs and lncRNAs. Currently, however, there is no known clinical research on fibrotic diseases that is based on epigenetics. This would be essential to identify not only new therapies but also predictive biomarkers for the diagnosis of fibrotic diseases, such as many autoimmune diseases. In an attempt to optimize the general application and effectiveness of therapies targeted against IL-17 subtypes and to clarify the multiple molecular pathways in which they appear to carry out their regulatory activity, it will be necessary to elucidate the immunological and genetic circumstances under which IL-17 becomes pro-fibrotic.

## Figures and Tables

**Table 1:** Collective table reporting the mechanisms of action and related references for IL-17 subtypes.

Cytokines	Cellular Sources	Target Cells	Functions	References
**IL-17A**	Th17, γδT, macrophages, epithelial cells, dendritic cells	Epithelial cells, endothelial cells, inflammatory cells	Stimulation of inflammatory cells, release of profibrotic factors, pulmonary fibrosis, primary biliary cirrhosis, EMT, activation of NF-κB	[58,59,60,61,62]
**IL-17B**	Neutrophils, Th17, γδT, macrophages, epithelial cells, dendritic cells, B cells	Neutrophils, macrophages, lymphocytes	Release of IL-6, IL-23, IL-1α, TNF-α, IL-1β, regulation of the T helper (Th)-mediated immune responses, lung fibrosis, EMT	[63,64,65,67]
**IL-17C**	CD4^+^ T cells, dendritic cells, macrophages, epithelial cells	Th17 cells, epithelial cells	Severe inflammatory response, NF-κB/Act1 activation, pro-inflammatory factors, pulmonary fibrosis	[68,69,70,71,72,73]
**IL-17D**	B lymphocytes, resting CD4^+^ T cells	Endothelial cells	Secretion of inflammatory factors, exacerbation of pulmonary fibrosis	[73,74]
**IL-17E**	Th2 cells, epithelial cells, endothelial cells, T cells, alveolar macrophages, dendritic cells, eosinophils, basophils	Alveolar epithelial cells	EMT program, pulmonary fibrosis, increased levels of IL-17E in the lungs of IPF patients, IL-13 release	[75,76,77]
**IL-17F**	Th17, γδT, macrophages, epithelial cells, dendritic cells	Tracheal epithelial cells, inflammatory cells, endothelial cells, fibroblasts	IL-6 and CXC chemokines, autoimmune and inflammatory diseases, profibrotic activity	[78,79,80]

**Table 2:** Positive or negative correlations in lncRNA/miRNA expression and IL-17 levels in fibrotic diseases or autoimmune fibrotic diseases.

lncRNA/miRNA	Effect on IL-17	Signalling Pathway	Fibrotic Diseases	References
miR-21	positive		liver fibrosis	[110,111]
lmiR-142-5p; miR-21	positive		autoimmune uveoretinitis	[112]
miR-182	negative		autoimmune uveoretinitis	[112]
lmiR-1266; lmiR-146	positive		psoriasis, also linked to RA	[113,114,115]
lmiR-21	negative	STAT3	RA	[116]
lmiR-145	negative		experimental autoimmune myastenia gravis	[106]
lmiR-132	negative		EAE	[124]
lmiR-20b	negative	RORγt; STAT3	MS/EAE	[122]
lmiR-873; lmiR-326	positive	A20; NF-kB; ets-1	MS/EAE	[123]
miR-326	positive	Ets-1 inhibition	MS/EAE	[125]
lncRNA-AK081284	positive		cardiac fibrosis	[117]
miRNA-129-5p	negative	collagen alpha-1	SSc	[120]
miR-125a-3p	negative		Lupus nephritis	[121]

RA: rheumatoid arthritis; EAE: experimental autoimmune encephalomyelitis; MS: multiple sclerosis; SSc: systemic sclerosis.

## Data Availability

Not applicable.
